# Chicken novel leukocyte immunoglobulin-like receptor subfamilies B1 and B3 are transcriptional regulators of major histocompatibility complex class I genes and signaling pathways

**DOI:** 10.5713/ajas.18.0561

**Published:** 2018-10-26

**Authors:** Anh Duc Truong, Yeojin Hong, Janggeun Lee, Kyungbaek Lee, Ha Thi Thanh Tran, Hoang Vu Dang, Viet Khong Nguyen, Hyun S. Lillehoj, Yeong Ho Hong

**Affiliations:** 1 Department of Animal Science and Technology, Chung-Ang University, Anseong 17546, Korea; 2 Department of Biochemistry and Immunology, National Institute of Veterinary Research, 86 Truong Chinh, Dong Da, Hanoi 100000, Vietnam; 3 Animal Biosciences and Biotechnology Laboratory, Agricultural Research Services, United States Department of Agriculture, Beltsville, MD 20705, USA

**Keywords:** Chicken, Leukocyte Immunoglobulin-like Receptor 1 (LILRB1), LILRB3, Major Histocompatibility Complex (MHC) Class I, Cytokines

## Abstract

**Objective:**

The inhibitory leukocyte immunoglobulin-like receptors (LILRBs) play an important role in innate immunity. The present study represents the first description of the cloning and structural and functional analysis of LILRB1 and LILRB3 isolated from two genetically disparate chicken lines.

**Methods:**

Chicken *LILRB1-3* genes were identified by bioinformatics approach. Expression studies were performed by transfection, quantitative polymerase chain reaction. Signal transduction was analyzed by western blots, immunoprecipitation and flow cytometric. Cytokine levels were determined by enzyme-linked immunosorbent assay.

**Results:**

Amino acid homology and phylogenetic analyses showed that the homologies of LILRB1 and LILRB3 in the chicken line 6.3 to those proteins in the chicken line 7.2 ranged between 97%–99%, while homologies between chicken and mammal proteins ranged between 13%–19%, and 13%–69%, respectively. Our findings indicate that LILRB1 and LILRB3 subdivided into two groups based on the immunoreceptor tyrosine-based inhibitory motifs (ITIM) present in the transmembrane domain. Chicken line 6.3 has two ITIM motifs of the sequence LxYxxL and SxYxxV while line 7.2 has two ITIM motifs of the sequences LxYxxL and LxYxxV. These motifs bind to SHP-2 (protein tyrosine phosphatase, non-receptor type 11) that plays a regulatory role in immune functions. Moreover, our data indicate that LILRB1 and LILRB3 associated with and activated major histocompatibility complex (MHC) class I and β2-microglobulin and induced the expression of transporters associated with antigen processing, which are essential for MHC class I antigen presentation. This suggests that LILRB1 and LILRB3 are transcriptional regulators, modulating the expression of components in the MHC class I pathway and thereby regulating immune responses. Furthermore, LILRB1 and LILRB3 activated Janus kinase2/tyrosine kinase 2 (*JAK2/TYK2*); signal transducer and activator of transcription1/3 (*STAT1/3*), and suppressor of cytokine signaling 1 genes expressed in Macrophage (HD11) cells, which induced Th1, Th2, and Th17 cytokines.

**Conclusion:**

These data indicate that LILRB1 and LILRB3 are innate immune receptors associated with SHP-2, MHC class I, β2-microglobulin, and they activate the Janus kinase/signal transducer and activator of transcription signaling pathway. Thus, our study provides novel insights into the regulation of immunity and immunopathology.

## INTRODUCTION

In mammals, leukocyte immunoglobulin-like receptors (LILRs) include 11 functional genes, which are classified as activating (LILRA1, 2, 4–6), inhibitory (LILRB1–5), and soluble (LILRA3). They are highly homologous in their extracellular regions but differ in their intracellular regions [1,2]. The leukocyte immunoglobulin (Ig)-like receptor subfamily B (LILRB) of humans was first described and cloned in 1997 [3] and acts as an important group of immunoreceptor tyrosine-based inhibitory motifs [ITIMs: I/V/L/S)XYXX(L/V)] containing receptor in their cytoplasmic tail. It belongs to a group of type I transmembrane glycoproteins with extracellular Ig-like domains that bind ligands and intracellular ITIMs [4,5]. The structure of LILRB groups consists of a signal peptide, two or four Ig-like domains, transmembrane domain and a long cytoplasmic tail with ITIMs [1,6]. Moreover, the LILRB groups are classified into group 1 (LILRB1 and LILRB2) and group 2 (LILRB3, LILRB4, and LILRB5). They consist of members that interact with the human leukocyte antigen (HLA) class I molecules and non-HLA ligands and are well-characterized and have been increasingly identified in the recent years [1,2,6].

These LILRB groups are expressed on various cell types such as macrophages, T cells, B cells, NK cells, and dendritic cells although the expression patterns are different between them [1,2,6]. ITIMs are activated by various phosphatases such as protein tyrosine phosphatase, non-receptor type 6 (PTPN6 or SHP-1), SHP-2 (PTPN11), or Src homology 2 domain-containing inositol phosphatase (SHIP) [1]. These phosphatases are capable of dephosphorylating multiple signaling-related molecules such as extracellular-regulated kinase (ERK), Janus kinase/signal transducer and activator of transcription (JAK/STAT), mitogen-activated protein kinase (MAPK) or nuclear factor kappa B subunit signaling pathway [7]. LILRB groups have been shown to control the activity of toll-like receptors (TLR) [8], antigen-presenting and cytokine production, thus demonstrating that the LILRB groups play an important role in the regulation of innate immune responses [8]. Moreover, the gene expression and polymorphisms of LILRBs have been reported to be associated with autoimmune and infectious diseases such as those caused by *Salmonella* [8], cytomegalovirus [9], and *Mycobacterium tuberculosis* [10], as well as rheumatoid arthritis [11], Alzheimer’s model [12], psoriatic arthritis [13], HIV/AIDS [14], and cancer [6]. Recently, some members of the Ig superfamily that may be involved in immune responses such as triggering receptor expressed on myeloid cells, cluster of differentiation 300, signal regulatory protein and chicken Ig-like receptors (CHIR)-A, CHIR-B, and CHIR-AB homologs were identified in chicken using bioinformatics approaches [15]. The studies of the chicken leukocyte immunoglobulin receptor (LIR) indicated that *LIR* genes are shown highly polymorphic locus harboring a variable number of *CHIR* genes and also play an important role in avian influenza infection in chicken [15]. However, pertinent information is lacking in chicken; for example, no data are available on the primary structure of LILRB1 and LILRB3, and the functions of those have not yet been investigated.

In this paper, therefore, we have identified novel *LILRB1* and *LILRB3* genes and described the expression and functional analysis of the variants of *LILRB1* and *LILRB3* genes in chicken, namely LILRB1R, −B1S, −B3R, and −B3S, which are isolated from two genetically disparate chicken lines in macrophage (HD11) cell lines. Our findings strongly suggest that the LILRB1 and LILRB3 associated with major histocompatibility complex (MHC) class I and non-classical β2-microglobulin (β2m) and controlled the constitutive signaling mediated by SHP-2 that regulates the JAK/STAT signaling pathway. We also described that LILRB1 and LILRB3 were expressed on HD11 cells and they modulated cytokines production.

## MATERIALS AND METHODS

### Bioinformatic analysis and identification of chicken *LILRB1* and *B3* genes

Our previous study [16,17] reported that more than 15,000 novel transcripts are expressed in the chicken lines 6.3 (R) and 7.2 (S), which associated with Marek’s disease (MD) resistance and shared the same MHC haplotype. These sequences were then used to search all the current chicken genome assemblies through the *Gallus_gallus-5.0 reference Annotation Release 103* nucleotide BLAST program (https://blast.ncbi.nlm.nih.gov/Blast.cgi). The chromosomal locations were clarified using the BLAST-Like Alignment Tool (BLAT) (http://asia.ensembl.org/Gallus_gallus/Info/Index) (Supplementary Table S1). The genomic DNA identified as encoding potential chicken *LILRB1* and *LILRB3* genes was then analyzed using GeneScan [18] to predict the coding DNA sequence (CDS) and protein domains. To confirm the sequences of two genetically disparate chicken lines, primers were designed using the Lasergene software (DNASTAR Inc. Madison, WI, USA) and synthesized by Genotech Co. Ltd. (Daejeon, Korea) (Supplementary Table S2). The polymerase chain reaction (PCR) products from 10 individual samples of chicken line 6.3 and line 7.2 were purified using the QIAQuick gel Extraction Kit (QIAgen, Hilden, Germany), and sub-cloned into the pCR2.1-TOPO vector (Invitrogen, Carlsbad, CA, USA), followed by transformation into *Escherichia coli* (*E. coli*) TOP10 (Invitrogen, USA) high-efficiency competent cells. Through blue-white screening, the positive clones were selected and sequenced by Genotech (Korea). Protein identification was conducted using the Expert Protein Analysis System (ExPASy; http://www.expasy.org/tools/) and the multiple sequence alignment was analyzed using the Lasergene software (DNASTAR Inc., USA). To draw a phylogenetic tree of the amino acid sequences of LILRB1 and LILRB3 groups, the neighbor-joining method with a bootstrap value of 1,000 in the MEGA6 program (https://www.megasoftware.net/) was used. Signal peptides were predicted by SignalP 4.1 Server v.4.1 (http://www.cbs.dtu.dk/services/SignalP/) and glycosylation motifs were predicted using NetOGlyc 4.0 Server (www.cbs.dtu.dk/services/NetOGlyc/). The Ig domains, transmembrane domain, and the cytoplasmic regions were predicted by InterPro v.56.0 (https://www.ebi.ac.uk/interpro/). The ITIMs were mapped to each peptide sequence using the established V/L/S/NxYxxL/V ITIM motif [4,5]. Full-length CDS of *LILRB1* and *LILRB3* genes were cloned in pCR2.1 (Invitrogen, USA) and excised from pCR2.1 using *NotI*/*Xbal* (Bioneer Corp, Daejeon, Korea), and ligated into the eukaryotic expression vector, pcDNA3-eGFP (Addgene, Cambridge, MA, USA), and then, followed by the transformation of *E. coli* BL21 (Invitrogen, USA). The positive clones were sequenced, and the structure and the ligand-binding site of the protein were determined by molecular replacement using the program RaptorX web server (http://raptorx.uchicago.edu/). To identify the function, the ligands of *LILRB1* and *LILRB3* genes were mapped and searched using the RCSB protein data bank (http://www.rcsb.org).

### Transfection into the macrophage (HD11) cell line

The chicken macrophage (HD11) cells [19] were transiently transfected with a pcDNA3-eGFP vector containing either the LILRB1R-, LILRB1S-, LILRB3R-, or LILRB3S-encoding sequence and also mock control with empty pcDNA3-eGFP vector using Lipofectamine 3000 transfection reagent (Invitrogen, USA), as per the manufacturer’s recommended protocols. A total of 1.0×106 cells per well in six-well plate (Thermo Scientific, Waltham, MA, USA) were transfected with 4.0 μg of plasmid using the Lipofectamine 3000 reagent and the transfected cells were harvested after 48 h for further analysis.

### Reagents

Mouse monoclonal anti-GFPuv/eGFP antibody was purchased from R&D Systems (Minneapolis, MN, USA). Mouse anti-chicken MHC class I-PE and mouse anti-chicken β2m-PE antibodies were purchased from Southern Biotech (Birmingham, AL, USA). Rabbit anti-chicken p-STAT1 (Ser^727^), anti-chicken p-STAT3 (Ser^727^), and anti-chicken p-JAK2 (Tyr^100^7/Tyr^1008^) were purchased from Santa Cruz Biotech (Dallas, TX, USA). Rabbit anti-chicken suppressor of cytokine signaling 1 (SOCS1), anti-chicken STAT1, anti-chicken STAT3 antibodies, horseradish peroxidase (HRP)-linked anti-rabbit secondary antibodies, and protein G–sepharose beads were purchased from Sigma-Aldrich (St. Louis, MO, USA). Rabbit anti-chicken p-SHP2 (Tyr^542^), rabbit anti-chicken JAK2, and rabbit anti-chicken TYK2 antibodies were purchased from Biorbyt (San Francisco, CA, USA). Rabbit anti-chicken glyceraldehyde-3-phosphate dehydrogenase (GAPDH) antibody was purchased from Abcam (Cambridge, MA, USA). Anti-His (C-Term)-HRP and Alexa Fluor 488 goat anti-rabbit IgG (H+L) secondary antibodies were purchased from Invitrogen (USA). Mouse monoclonal anti-chicken interferon (IFN)-γ antibody, mouse monoclonal anti-chicken interleukin (IL)-17A antibody, mouse monoclonal anti-chicken IL-12p40 antibody and recombinant versions of these proteins (kindly provided by Dr. Hyun S. Lillehoj, USDA), EZ-Link Sulfo-NHS-LC-Biotin, Goat anti-mouse IgG secondary antibody linked HRP conjugate, and HRP-conjugated Streptavidin were purchased from Thermo Scientific (USA).

### Flow cytometry analysis

The cells were incubated in a flow cytometer buffers (10% fetal calf serum), 15 mM 4-(2-hydroxyethyl)-1-piperazineethanesulfonic acid, and 2 mM ethylenediaminetetraacetic acid [EDTA] in phosphate buffered saline [PBS]) and primary antibodies were added to 1.0×10^6^ cells and then, kept for 30 min on ice. To evaluate the LILRB1- and LILRB3-eGFP binding with MHC class I and β2m, anti-MHC class I-PE and anti-β2m-PE antibodies were incubated with the cells as described above, followed by a single wash with the flow cytometer buffers. For assessing LILRB1- and LILRB3-eGFP constitutive signaling mediated by SHP-2, anti-SHP-2 antibody was added and incubated for 30 min on ice, followed by a single wash with the flow cytometer buffers before adding Alexa Fluor 488-conjugated anti-rabbit secondary antibody (Invitrogen, USA). The control groups were incubated with goat anti-chicken IgG-PE or rabbit IgG only (data not show). Flow cytometry analysis was performed with BD FACS Aria II cell analyzer (BD Biosciences, San Jose, CA, USA). Data were acquired with BD FACS Diva Version 6.1.3 and analyzed using FlowJo 7.6.1. Forward and side-scatter gating removed contaminants such as cell debris.

### Immunoprecipitation and western blotting

Cells were washed twice with ice-cold PBS, harvested in ice-cold RIPA buffer (50 mM Tris-HCl pH 7.5, 150 mM NaCl, 2 mM EDTA, 100 mM sodium fluoride, 0.1% [w/v] sodium dodecyl sulfate [SDS], 0.5% [w/v] sodium deoxycholate, 1% Triton X-100, 10 mM sodium pyrophosphate, and 10 mM sodium orthovanadate) containing complete EDTA-free protease inhibitor cocktail (Thermo Scientific, USA), then gently lysed for 30 min at 4°C, and centrifuged at 13,000 g for 15 min at 4°C. The concentration of protein was evaluated using the Coomassie protein assay kit (Thermo Scientific, USA) in microplates as per the manufacturer’s instructions. The protein (100 μg) was incubated with the anti-eGFP mAb or anti-GAPDH antibody at 4°C overnight and then added to 50 μL of protein G–Sepharose (Invitrogen, USA) for 2 h. The immunoprecipitates were then washed three times in RIPA buffer and solubilized in 2×SDS-polyacrylamide gel electrophoresis (PAGE) sample buffer. Samples were electrophoresed on Tris-glycine SDS-PAGE gels, and the proteins were transferred on to polyvinylidene fluoride membranes (GE Healthcare, Rydalmere, Australia). Membranes were blocked with 5% non-fat milk (Thermo Scientific, USA) or 3% bovine serum albumin (BSA) (Sigma-Aldrich, USA) in PBS pH 7.4 containing 0.05% Tween 20 (PBST) for 2 h at room temperature (RT, 25°C), washed with PBST, and incubated with anti-MHC class I, anti-β2m or anti-SHP-2 antibody or signaling antibodies overnight at 4°C and the relevant secondary antibody in 2% non-fat milk or 0.5% BSA in PBST for 2 h at RT. Finally, the membranes were developed using Western Lightning ECL Plus (Thermo Scientific, USA) and Hyperfilm (GE Healthcare, Australia).

### Enzyme-linked immunosorbent assay

We coated a 96-well plate (Nunc MaxiSorp, Nunc, Wiesbaden, Germany) with dilutions (1:500) of monoclonal anti-IFN-γ, anti-IL-12p40, or anti-IL-17A antibodies for 7 d at 4°C as previously described [20]. Next, the plates were blocked with 5% non-fat milk for 2 h at 25°C, the plates were incubated overnight at 4°C with the cell supernatant, or different dilutions of the recombinant IFN-γ, IL-17A, and IL-12p40. Following incubation with a biotinylated monoclonal IFN-γ, IL-17A, or IL-12p40 antibody, HRP-conjugated Streptavidin was added. The plates were washed three times with PBST at each step. Then, 3,3′,5,5′-tetramethylbenzidine (Thermo Scientific, USA) was used as the chemiluminescent substrate and the luminescence was measured using a Hybrid Microplate Reader (Epoch, BioTek Instruments, Inc, Winooski, VT, USA).

### Quantitative real-time polymerase chain reaction

After 48 h of transfection with a pcDNA3-eGFP vector containing either the LILRB1R, −B1S, −B3R, or −B3S encoding sequence and a mock control, RNA was extracted using the TRIzol kit (Invitrogen, USA) as per manufacturer’s instructions. Next, 2 μg total RNA was reverse transcribed to cDNA by using Maxima First Strand cDNA Synthesis Kit (Thermo Scientific, USA), according to manufacturer’s protocols. Quantitative PCR (qPCR) was performed using Light Cycler 96 Real-time PCR System (Roche Diagnostics, Indianapolis, IN, USA) in a 20-μL reaction mixture containing 1 μL cDNA, 10 μL 2× FastStart Universal SYBR Green Master Mix (Roche Diagnostics, USA), and 10 pmol each of forward and reverse primers of genes (Supplementary Table S2). The primers were designed using the Lasergene software (DNASTAR Inc., USA) and were synthesized by Genotech Co. Ltd. (Korea). Thermal conditions for performing qPCR are as follows: initial incubation at 95°C for 5 min; 40 cycles of denaturation at 95°C for 30 s, annealing at 50°C to 55°C for 30 s, and extension at 72°C for 30 s; and termination by final incubation at dissociation temperatures 95°C (10 s), 65°C (60 s), 97°C (1 s), and 37°C (30 s). Genes expression were quantified after normalization with the chicken *GAPDH* gene and were calculated using the 2^−ΔΔCt^ method.

### Bioactivity assays

To determine the cytotoxicity of LILRB1R, −B1S, −B3R, and −B3S linked eGFP vector and empty vector transfected into HD11 cell line after 48 h transfection, cell proliferation and NO production assay was performed in 96-well plates according to well-established protocols [20]. To measure the nitrite content, 100 μL of the culture medium was incubated with 100 μL of Griess reagent (Sigma-Aldrich, USA) at RT for 10 min. Then, the absorbance was measured at 540 nm using a microplate reader as described [20]. The nitrite content was calculated based on a standard curve constructed with NaNO_2_. Cell proliferation was determined with the cell counting Kit-8 (CCK-8) assay according to the manufacturer’s protocol as described [20] (Dojindo Molecular Technologies, Inc., Mashikimachi, Kumamoto, Japan). Lipofectamine 3000 transfection reagent and culture medium were used as controls.

### Statistical analysis

Data are represented as the mean±standard error of the mean of three independent experiments for each group (n = 3) and were analyzed with the SAS 9.4 statistical program (SAS Institute Inc., 100 SAS Campus Drive Cary, NC, USA). Comparison between the experimental groups was carried out using Duncan’s multiple comparison method. Differences were considered significant when p<0.05.

## RESULTS

### Identification of potential chicken LILRB1 and LILRB3

**Figure 1:**
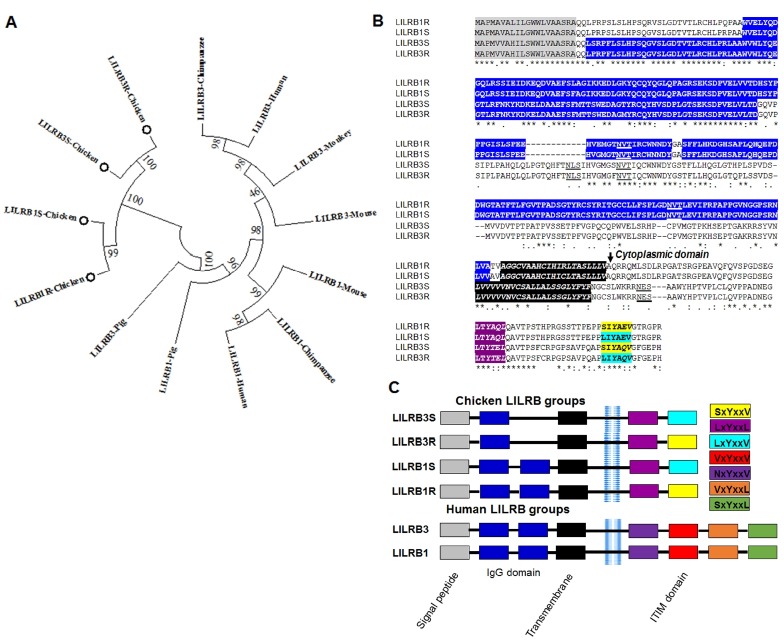
Genetic analysis of *LILRB1* and *LILRB3* genes identified from two genetically disparate chicken lines. (A) Phylogenetic tree indicating the relationship between chicken *LILRB1* and *LILRB3* amino acid sequences and those of other vertebrates; (B) Multiple alignment of the predicted *LILRB1* and *LILRB3* genes from two genetically disparate chicken lines was performed using the ClustalX program. Shown is the result of the amino acid sequence alignment of chicken *LILRB1* and *LILRB3* genes together with human *LILRB1* and *LILRB3* genes. Signal peptide, Ig domain, transmembrane, and cytoplasmic domain are indicated in dark, blue, black, and no color, respectively. The N-linked glycosylation sites are underlined; (C) Secondary structures of chicken *LILRB1* and *LILRB3* genes compared to those of the known human *LILRB1* and *LILRB3* genes. *LILRB*, leukocyte immunoglobulin-like receptors.

Based on the RNA-Seq results from two genetically disparate Necrotic Enteritis (NE)-afflicted chicken lines [16,17], we identified 11 open reading frames (ORFs) in our library as containing potential chicken *LILRB1* and *LILRB3* genes. Each of these ORFs was then analyzed by GeneScan and BLAT to obtain the predicted CDS and the corresponding protein sequence, which were then aligned to domains using protein BLAST to identify the Ig domains. All 11 ORFs were located on chromosome Un-random from 3,559,608 to 63,671,960 positions. Of the 11 ORF sequences, we considered 4 ORFs sequence groups, which contain Ig domains and showed different sequences between the two chicken lines. Then, we evaluated the expression levels of *LILRB1R*, −*B1S*, −*B3R*, and −*B3S* transcripts in both the NE-induced chicken lines, and the results showed that the expression of *LILRB1* and *LILRB3* transcripts in the chicken line 6.3 was higher than that of line 7.2 although the expression of these transcripts in both the NE-induced lines was higher than that of the control (Supplementary Table S1). Next, the novel mRNA transcripts of *LILRB1R*, −*B1S*, −*B3R*, and −*B3S* were amplified by RT-PCR, cloned, and sequenced for 10 individual samples from each chicken line. Nucleotide and amino acid sequence analyses showed that the identity of LILRB1 or LILRB3 in 10 samples of each chicken line was 100% (data not shown). Analysis of the *LILRB1R*, −*B1S*, −*B3R*, and −*B3S* nucleotide sequences showed a 30% to 38% similarity with mammals such as human, mouse, monkey, chimpanzee, and pig (data not shown). Multiple alignments of each predicted LILRB1 and LILRB3 protein on line 6.3 to those proteins on line 7.2 showed that the amino acids had an identity and similarity range of 97% to 99% (Table 1). Comparison of amino acid identities and similarities between LILRB1 and LILRB3 in chicken to mammals ranked between 13% to 19% and 13% to 69%, respectively (Table 1). Based on amino acid similarities, phylogenetic analysis placed the novel receptors together with the members of the mammalian *LILRB1* and *LILRB3* genes that were themselves clustered using the neighbor-joining bootstrap consensus trees inferred from 1,000 replicates (Figure 1A). The results indicated that these 4 receptors are closely associated with the *LILRB1* and *LILRB3* genes of other species. Specifically, it can be concluded that the LILRB1R, −B3R, and LILRB1S, −B3S amino acids isolated in chicken line 6.3 and line 7.2, respectively, are homologous based on the high degree of sequence similarity (Figure 1A). From these results, the newly identified transcripts from the two genetically disparate chicken lines can be classified as chicken LILRB1R, −B1S, −B3R, and −B3S.

### Structure and potential ligand binding of novel *LILRB1/3* genes

Overall, the secondary structure of chicken LILRB1 and LILRB3 proteins included the signal peptide, Ig domains, transmembrane, cytoplasmic domain, and two ITIM motifs (Figure 1B). Based on the Ig domain structure, we also divided these genes into 2 subgroups as group 1 (LILRB1R and LILRB1S) and group 2 (LILRB3R and LILRB3S). Group 1 contains 2 Ig domains (W^54^-Y^147^ and S^150^-A/V^231^) and two N-glycosylations (N^137^ and N^207^) whereas group 2 contains one Ig domain (L^23^-D^116^) and three N-glycosylations (N^138^, N^148^, and N^268^) (Figure 1B). Based on the presence or absence of ITIMs, the signaling domains found in the chicken *LILRB1* and *LILRB3* genes are different than those in humans. The human *LILRB1* and *LILRB3* genes have 4 ITIMs present in the LILRB1 and LILRB3 receptors: NxYxxV, VxYxxV, VxYxxL, and SxYxxL, whereas the chicken *LILRB1* and *LILRB3* genes contain 2 ITIMs, one of which, LxYxxL is common to all receptors (Figure 1C). It is suggested that human *LILRB1* and *LILRB3* genes might differ in their capacity of their ligand binds and regulate immune responses compared to chicken *LILRB1* and *LILRB3* genes. On the other hand, the second ITIMs motifs were different between *LILRB1* and *LILRB3* genes isolated from the two genetically disparate chicken lines. The SxYxxV motif appears in the resistant chicken lines (LILRB1R, −B3R) while LxYxxV in the susceptible lines (LILRB1S, −B3S) (Figure 1C). This suggests that the *LILRB1* and *LILRB3* genes contribute differently to cellular regulation and innate immunity in resistant and susceptible chicken lines. Moreover, in humans, it is reported that the ITIMs motif also determined the binding of the cytosolic tyrosine kinases SHP-1 and SHP-2 [4]. Thus, the results suggest that the ITIMs motif in the two genetically different chicken lines may be associated with tyrosine kinases and regulation of signaling pathways. Consequently, we concluded that our newly identified transcripts are significantly different in the two genetically disparate chicken lines. Therefore, it is suggested that chicken LILRB1 and LILRB3 proteins are similar to those in humans in structure and function and may differ only in their ligand binding sites.

Identification of ligands for *LILRB1* and *LILRB3* genes is a key step in clarifying the biology of LILRB family genes and understanding the function of these genes. The ligands for LILRB3 is not clear in humans. Moreover, LILRB1 has multiple ligands that bind with MHC class I [2]. Using the RaptorX web server program, we screened candidate ligands of LILRB1R, −B1S, −B3R, and −B3S, and identified their function. The ligand of *LILRB1* and *LILRB3* genes were mapped and searched in the RCSB protein data bank and also in previously reported literature. The results showed that *LILRB1* and *LILRB3* genes have at least two ligands each and most of the ligand candidates for *LILRB1* and *LILRB3* genes bound MHC-class I and β2m antigen (Table 2, Supplementary Figure S2). Comparison of the *LILRB1* and *LILRB3* genes between the two genetically disparate chicken lines showed that both genes had different ligand-binding residues. Therefore, this suggests that *LILRB1* and *LILRB3* genes may be binding MHC class I, and β2m differently in the two genetically disparate chicken lines and the binding might also differ between *LILRB1* and *LILRB3* genes.

### MHC-class I and β2-microglobulin heavy chain binds to LILRB1 and LILRB3

To confirm the efficiency of eGFP-linked with either LILRB1R, −B1S, −B3R, and −B3S and mock control (empty eGFP vector), these constructs were transfected into the HD11 cell line. First, we determined the transfection efficiency in LILRB1 or LILRB3-transfected HD11 cell line by fluorescent microscopy and fluorescence-activated cell sorting (FACS) analysis (Supplementary Figure S2A). The transfection efficiency was >90% as determined by FACS analysis (Supplementary Figure S2A). Finally, the expressed LILRB1 and LILRB3 proteins were determined by western blotting. As shown in Supplementary Figure S2B, a single band was detected by anti-eGFP mAb in the eGFP-linked LILRB1 lane and in the LILRB3-transfected HD11 cell line. Taken together, these data demonstrate that LILRB1 and LILRB3 proteins were constitutively expressed in the transfected HD11 cell line (Supplementary Figure S2B). Moreover, cytotoxicity analysis showed that the proliferation of chicken HD11 cells after transfection was not significantly inhibited or enhanced (Supplementary Figure S2B) and the concentration of NO production was higher in LILRB1- or LILRB3-eGFP group than in the control group (Supplementary Figure S2B). The results indicated that chicken *LILRB1* and *LILRB3* genes induced the intracellular production of reactive oxygen species in the form of NO.

**Figure 2:**
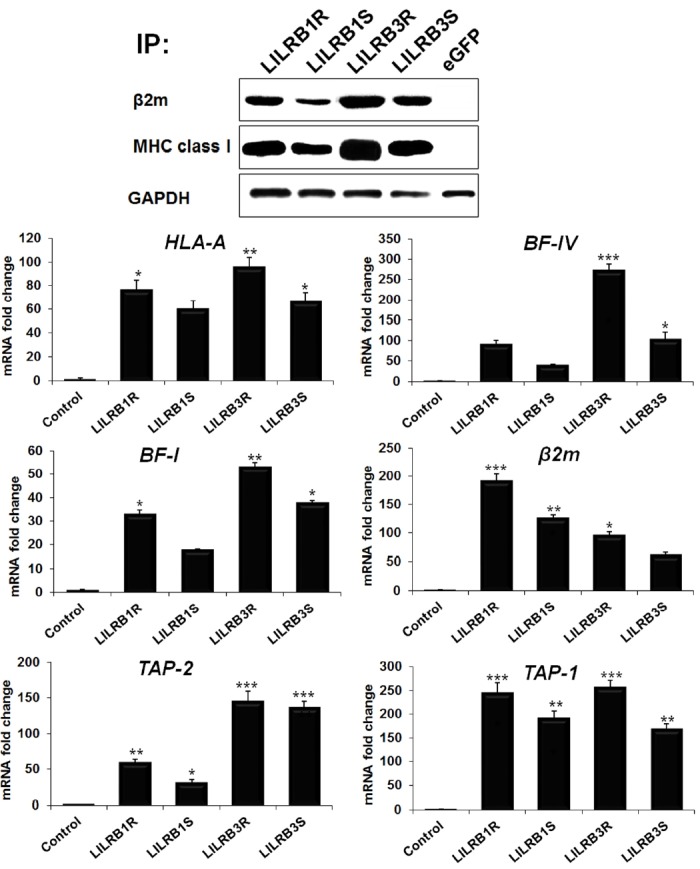
LILRB1 and LILRB3 glycoprotein associated with MHC class I and β2m. Top panel, HD 11 cells transfected with *LILRB1* and *LILRB3* genes from two genetically disparate chicken lines were lysed and then immunoprecipitated with the MHC class I and β2m mAb, then separated by sodium dodecyl sulfate-polyacrylamide gel electrophoresis and immunoblotted with a secondary antibody. Bottom panel, Changes in mRNA levels of the MHC class I family and its related genes in *LILRB1*- and *LILRB3*- transfected cells detected by quantitative reverse-transcription polymerase chain reaction. *LILRB*, leukocyte immunoglobulin-like receptors; MHC, major histocompatibility complex. Data are presented as the mean±standard error of the mean (n = 3) of three independent experiments: * p<0.05, ** p<0.01, and *** p<0.001.

In humans, some researchers demonstrated that LILRB family genes could bind with MHC class I and β2m [21]. As predicted, GFP-linked LILRB1 and LILRB3 showed a strikingly different pattern of gene expression compared with GFP alone. Our quantitative real-time PCR analysis in cells transfected with GFP-linked *LILRB1* and *LILRB3* genes and mock control showed that the *LILRB1* and *LILRB3* genes induced the expression of various members of the MHC class I (*HLA-A*, *BF-I*, and *BF-IV* [leukocyte antigens A, avian MHC class I alpha chain 1 and avian MHC class I alpha chain 2]) family as well as other genes involved in class I antigen presentation and processing, such as *β2m*, transporters associated with antigen processing 1 (*TAP1* gene), and *TAP2* (Figure 2). The result indicated that upregulation of MHC class I family genes and related genes by LILRB1R and −B3R activation was greater than that by LILRB1S and −B3S (Figure 2). Particularly, the expression of *β2m* mRNA was markedly upregulated in the HD11 cell line by 191.5-, 125.9-, 96.1-, and 61.2-fold by LILRB1R, −B1S, −B3R and −B3S, respectively. The expression levels of *BF-I*, *BF-IV*, *HLA-A*, *TAP1*, and *TAP2* genes were also highly upregulated due to LILRB3R activation by 53.0-, 272.4-, 95.3-, 256.1-, and 144.6-fold, respectively (Figure 2). Moreover, immunoprecipitation analysis demonstrated that LILRB1 and LILRB3 proteins were bound by MHC class I and β2m (Figure 2). We confirmed that the LILRB1R, −B1S, −B3R, and −B3S immunoprecipitates contained around 42- and 14-kDa size proteins corresponding to anti-MHC class I and β2m, respectively (Figure 2). The amount of LILRB1R, −B1S, −B3R, and −B3S immunoprecipitated with MHC class I was higher than that with β2m. Moreover, the amount of LILRB1R and −B3R precipitated with MHC class I and β2m was higher than LILRB1S and −B3S precipitated with MHC class I and β2m in macrophage cells, suggesting that the MHC class I and β2m associated with LILRB1R, −B1S, −B3R, and −B3S with different binding affinities (Figure 2).

**Figure 3:**
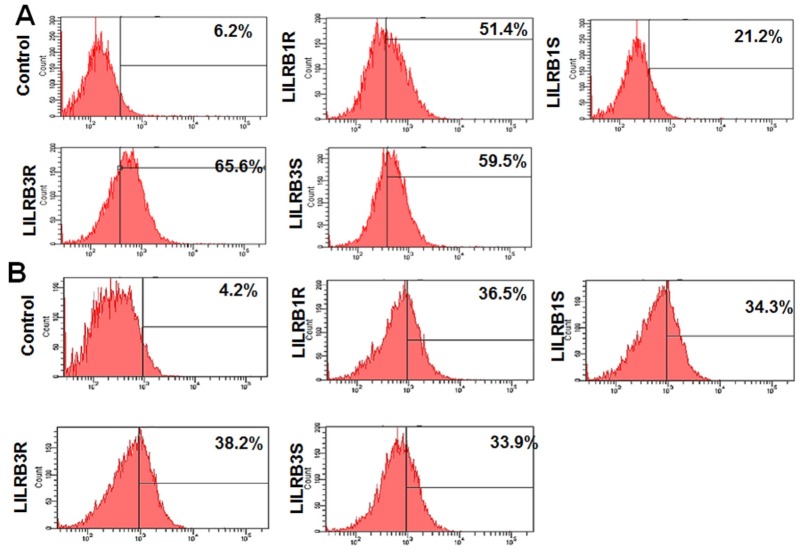
MHC class I and β2m mAb bind to *LILRB1* and *LILRB3* gene products from two lines. HD11 cells transfected with eGFP only or eGFP-linked constructs of LILRB1R, −B1S, −B3R, and −B3S stained with MHC class I (A) or β2m (B) labeled with phycoerythrin. Binding was assessed by fluorescence-activated cell sorting analysis. The resistant (line 6.3) and susceptible (line 7.2) lines are indicated as R and S, respectively. MHC, major histocompatibility complex; *LILRB*, leukocyte immunoglobulin-like receptors; eGFP, enhanced green fluorescent protein.

In addition, flow cytometry analysis using MHC class I- and β2m-specific antibodies confirmed an increase in MHC class I surface protein expression in *LILRB1* and *LILRB3* gene transfected cells. When the expression of *LILRB1* and *LILRB3* genes was upregulated, MHC class I and functionally related genes were expressed more and this increase in expression was higher in the case of the resistant chicken line 6.3 than in the susceptible chicken line 7.2 (Figure 3). In particular, LILRB1R, −B1S, −B3R and −B3S associated with MHC-I increased by 51.4%, 21.2%, 65.6%, 59.5%, respectively, and those associated with β2m by 36.5%, 34.3%, 38.2%, and 33.9%, respectively (Figure 3). In summary, these data indicated that *LILRB1* and *LILRB3* genes induce the expression of MHC class I and related genes involved in MHC class I antigen presentation and the level of binding with MHC class I and related genes was higher in line 6.3 than in line 7.2 and differed between the *LILRB1* and *LILRB3* genes.

### SHP-2 associated with phosphorylated LILRB1 and LILRB3 molecules

**Figure 4:**
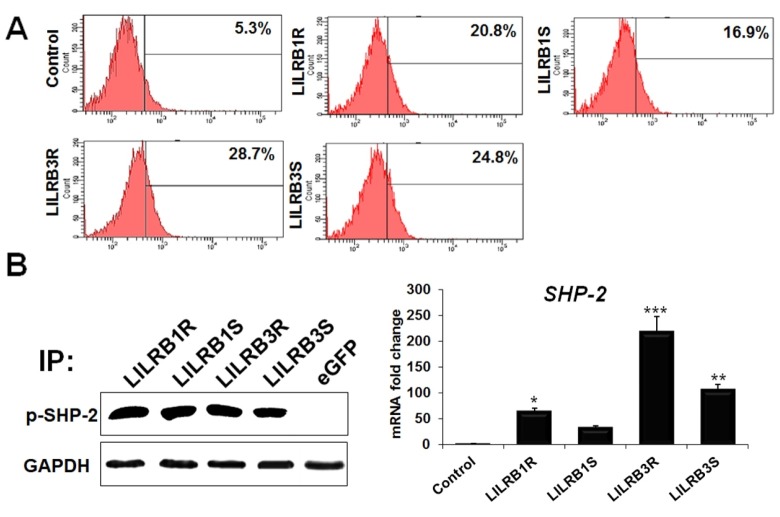
LILRB1R, −B1S, −B3R and −B3S glycoproteins associated with SHP-2. (A) Binding was assessed by fluorescence-activated cell sorting analysis. HD11 cells transfected with eGFP or eGFP-tagged constructs of LILRB1R, −B1S, −B3R, and −B3S stained with SHP-2 mAb and then by Alexa Fluor 488 labeled anti-rabbit secondary antibody. (B) Immunoprecipitation analysis of LILRB1 and LILRB3 associated with SHP-2 (Left) and changes of SHP-2 mRNA levels in cells transfected with LILRB1 and LILRB3 were detected by quantitative reverse-transcription polymerase chain reaction. LILRB, leukocyte immunoglobulin-like receptors; SHP-2, Tyrosine-protein phosphatase non-receptor type 11; HD11, macrophage cells; eGFP, enhanced green fluorescent protein. Data are presented as the mean±standard error of the mean (n = 3) of three independent experiments: * p<0.05, ** p<0.01, and *** p<0.001.

The tyrosine phosphatase SHP-2 associates with phosphorylated LILRB groups in B cells, macrophage cells and NK cells [1]. The presence of two ITIMs sequences located within the cytoplasmic region of *LILRB1* and *LILRB3* genes suggests that they could associate with SHP2 signaling pathways. To determine whether *LILRB1* and *LILRB3* genes are associated with SHP-2, we examined their tyrosine phosphorylation profiles after transfecting LILRB1-eGFP and LILRB3-eGFP into the HD11 cell line as shown in Figure 4. Firstly, flow cytometry analysis using a p-SHP-2 antibody was performed to confirm an increase of p-SHP-2 expression in cells transfected with *LILRB1* and *LILRB3* genes. The expression levels of p-SHP-2-associated LILRB1R, −B1S, −B3R and −B3S increased in the macrophage cell line by 20.8%, 16.9%, 28.7%, and 24.8%, respectively (Figure 4A). It is interesting that the levels of p-SHP-2-associated LILRB1 and LILRB3 were higher in line 6.3 than in line 7.2 (Figure 4A). Furthermore, when HD11 cell lysates were immunoprecipitated with anti-GFP mAb and immunoblotted with an anti-pSHP-2 antibody, approximately 66 kDa size proteins were detectable in the anti-pSHP-2 immunoprecipitates, but not in the mock control. These sizes corresponded to those of LILRB1R, −B1S, −B3R, and −B3S (Figure 4B). These results demonstrate that tyrosine phosphorylation is essential for SHP-2 binding to LILRB1R, −B1S, −B3R, and −B3S. Moreover, quantitative real-time PCR analysis confirmed the expression levels of *SHP-2* in cells transfected with GFP-linked *LILRB1*, *LILRB3* genes and GFP alone (Figure 4B). The *SHP-2* mRNA expression was highly upregulated in LILRB3R-transfected HD11 cell line compared to the mock control and was significantly upregulated by 63.7-, 218.7-, 106.2-fold corresponding to LILRB1R, −B3R, and −B3S, respectively (Figure 4B). Thus, we concluded that the expression of SHP-2 in LILRB1R- and −B3R-transfected macrophage cell line was higher than LILRB1S and −B3S-transfected macrophage cell line. Also, LILRB1 and LILRB3 were constitutively tyrosine phosphorylated and associated with SHP-2, which plays an important role in the modulation of signaling pathways in the chicken macrophage cell line.

### LILRB1 and LILRB3 promote the phosphorylation of signaling transduction molecules STAT and JAK

**Figure 5:**
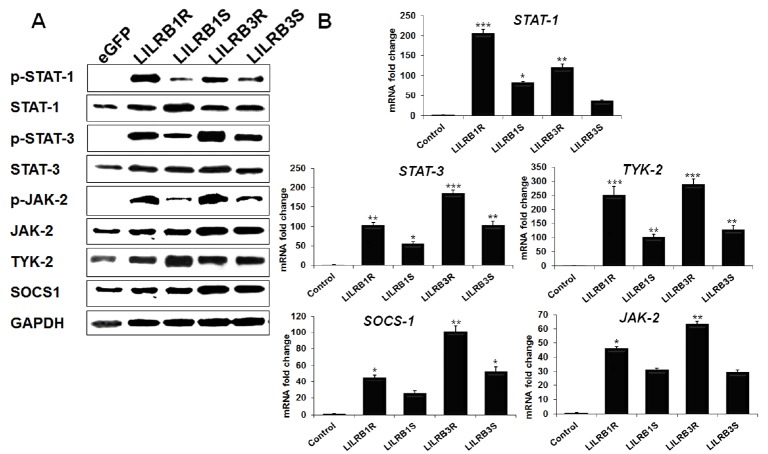
LILRB1R, −B1S, −B3R and −B3S glycoprotein regulated JAK/STAT signaling pathway. (A) Western blotting results of JAK2/TYK2, STAT1/3, and SOCS1 after LILRB1 and LILRB3 transfection in HD11 cell line. (B) Changes in mRNA levels for *JAK2/TYK2*, *STAT1/3*, and *SOCS1* genes after *LILRB1* and *LILRB3* gene transfection in HD11 cell line were detected by quantitative reverse-transcription polymerase chain reaction. *LILRB*, leukocyte immunoglobulin-like receptors; HD11, macrophage cell; *JAK2/TYK2*, Janus kinase2/tyrosine kinase 2; *STAT1/3*, signal transducer and activator of transcription1/3; *SOCS1*, suppressor of cytokine signaling 1. Data are presented as the mean±standard error of the mean (n = 3) of three independent experiments: * p<0.05, ** p<0.01, and *** p<0.001.

The JAK/STAT signaling pathway was investigated to determine the effect of the *LILRB1* and *LILRB3* genes on signaling pathways. JAK/STAT pathway plays a pivotal role in many important biological responses related to the immune response [22]. Here, we observed that STAT1 (p-Ser^727^), STAT3 (p-Ser^727^), and JAK2 (p-Tyr^1007^/Tyr^1008^) were phosphorylated in *LILRB1* and *LILRB3* genes-transfected HD11 cell line, while un-phosphorylated (STAT1/3, JAK2, TYK2, and SOCS1) proteins were also detected in *LILRB1* and *LILRB3* genes-transfected HD11 cell line by western blotting. The level of signaling proteins was higher in LILRB1R- and −B3R- than in −B1S- and −B3S-transfected cell lines (Figure 5A), suggesting that *LILRB1* and *LILRB3* genes in two genetically disparate chicken lines can differently activate the JAK/STAT signaling pathway.

The transcriptional expression level of the signaling pathway genes was evaluated by qRT-PCR and the results are shown in Figure 5B. The *STAT1/3* mRNA expression was highly upregulated in LILRB1R-transfected cells by 205.5 and 103-fold and in LILRB3R-transfected cells by 119.7- and 184.4-fold, respectively. On the other hand, *STAT1/3* expression levels of LILRB1S-transfected cells decreased by 82.4-, 55.5-fold and 36.1-, 102.6-fold in LILRB3S-transfected cells, respectively (Figure 5B). Moreover, *JAK2* and *TYK2* mRNA were more highly expressed in LILRB1R-, LILRB3R-transfected HD11 cells compared to LILRB1S- and LILRB3S-transfected cells. The expression of *JAK2* and *TYK2* mRNA was markedly upregulated by 63.1-, 286.2-fold, and 29.3-, 128.7- fold in LILRB3R- and −B3S-transfected HD11 cells, respectively (Figure 5B). We also evaluated the expression of *SOCS1* by Western blot and qRT-PCR analysis (Figure 5B). SOCS1 protein was strongly detected in *LILRB3R* gene-transfected cells and qRT-PCR analysis showed strong upregulation of *SOCS1* mRNA in LILRB1R- and LILRB3R-transfected cells (Figure 5). In particular, *SOCS1* mRNA expression was strongly upregulated by 100.8-fold in the LILRB3R-transfected HD11 cells (Figure 5B). These results indicate that LILRB1 and LILRB3 associate with the JAK/STAT signaling pathway and differentially regulate the immune response depending on the *LILRB* gene isolated from the two genetically disparate chicken lines.

### *LILRB1* and *LILRB3* upregulate cytokine genes expression

**Figure 6:**
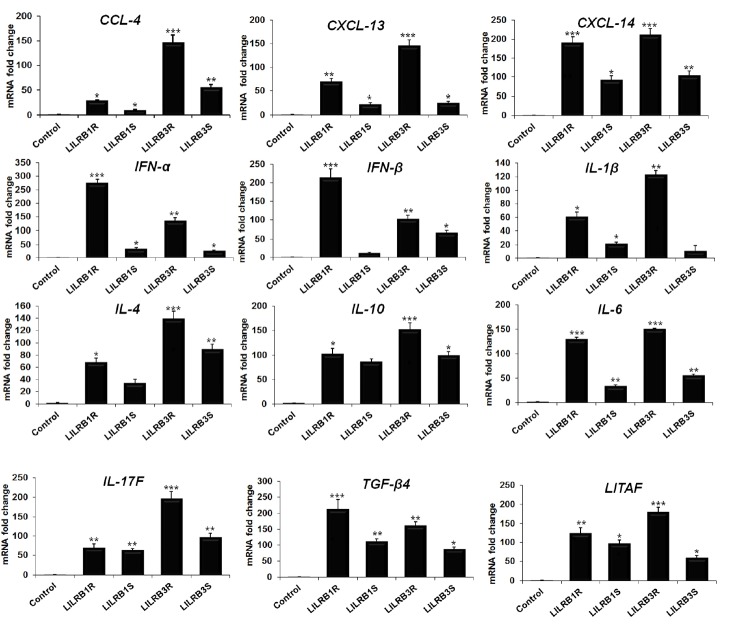
Changes in expression levels of cytokines caused by *LILRB1R*, *−B1S*, *−B3R*, and *−B3S* genes in transfected HD11 cell line. *LILRB*, leukocyte immunoglobulin-like receptors; HD11, macrophage cells; CCL, C-C motif chemokine ligand; CXCL, C-X-C motif chemokine ligand; IFN-γ, interferon-γ; IL, interleukin; TGF, transforming growth factor beta; LITAF, lipopolysaccharide-induced tumor necrosis factor-alpha factor. Data are presented as the mean±standard error of the mean (n = 3) of three independent experiments: * p<0.05, ** p<0.01, and *** p<0.001.

To determine the influence of *LILRB1* and *LILRB3* genes isolated from two chicken lines on the cytokine genes expression in HD11 cell line, qRT-PCR was performed. The expression levels of chemokines, C-C motif chemokine ligand (*CCL*)-*4*, C-X-C motif chemokine ligand (*CXCL*)-*13*, and *CXCL-14* mRNA were significantly increased, and particularly they were highly expressed in LILRB3R- transfected cells. Also, the levels of expression of JAK/STAT pathway-associated genes were highly upregulated in LILRB1R, −B3R-transfected cells than in −B1S and −B3S-transfected cells (Figure 6). The Th1-type cytokines, *IFN-α*, *IFN-β*, *IL-1β*, and *IL-6* mRNA were dramatically and differentially upregulated by the *LILRB1* and *LILRB3* genes transfection. Particularly, expression of *IFN-α* and *IFN-β* mRNA were increased by 273.4- and 213.3-fold, respectively, in LILRB1R-transfected cells. On the other hand, the expression of *IL-1β* and *IL-6* mRNA was upregulated by 123.3- and 149.6-fold, respectively, in LILRB3R-transfected cells (Figure 6). The effects of these transfected genes on the Th2-type cytokines (*IL-4* and *IL-10*), Treg cytokine (transforming growth factor beta [*TGF-β*]*4*), and proinflammatory mediator (lipopolysaccharide-induced tumor necrosis factor-alpha factor [*LITAF*]) were also examined. *TGF-β4* mRNA was strongly upregulated by 211.5-fold in LILRB1R-transfected cells; and *IL-4*, *IL-10*, and *LITAF* mRNA were significantly increased by 138.8-, 151.0-, 178.3-fold, respectively, after transfection with LILRB3R (Figure 6). Moreover, the expression of Th17-type cytokine gene (*IL-17F*) was also increased by 194.5-fold in LILRB3R-transfected cells (Figure 6). Therefore, these results suggest that *LILRB1* and *LILRB3* genes induce chemokines, Th1/2/17 cytokines production *in vitro* in activated macrophage cell lines. Also, the expression of genes involved in cytokines production was higher in the resistant chicken line 6.3 than those in the susceptible line 7.2.

### LILRB1 and LILRB3 induce cytokines production

**Figure 7:**
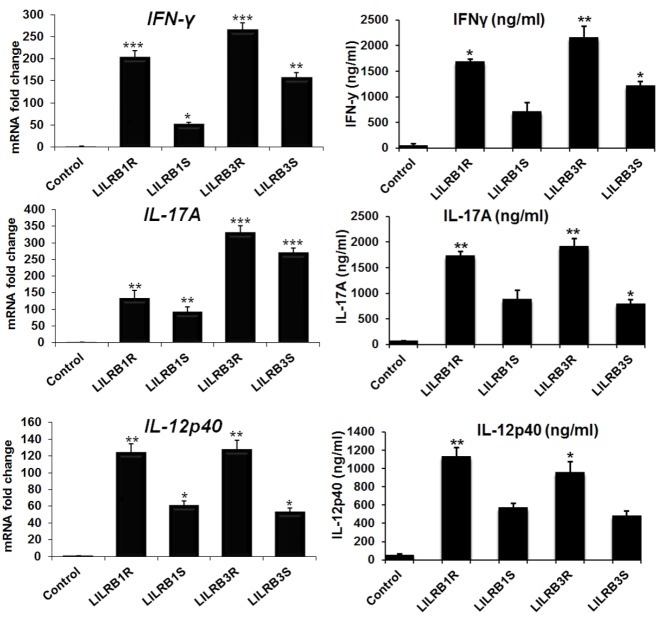
Changes in expression levels of cytokine production caused by LILRB1R, −B1S, −B3R, and −B3S in transfected HD11 cell line. LILRB, leukocyte immunoglobulin-like receptors; HD11, macrophage cells; IFN-γ, interferon-γ; IL, interleukin. Data are presented as the mean±standard error of the mean (n = 3) of three independent experiments: * p<0.05, ** p<0.01, and *** p<0.001.

The expression level of IFN-γ mRNA was dramatically increased in the LILRB1R-, −B1S-, −B3R-, and −B3S-transfected HD11 cell lines compared to the mock control by 203.6-, 51.9-, 264.4-, and 157.6-fold, respectively (Figure 7). To determine if the increased *IFN-γ* mRNA expression is mirrored by an increased IFN-γ protein in the supernatant, we analyzed the IFN-γ protein expression in the supernatant of LILRB1- and LILRB3-transfected HD11 cell lines and mock controls by enzyme-linked immunosorbent assay. The results revealed significantly higher IFN-γ protein levels by 1,691.7, 711.1, 2,160.2, and 1,219.82 ng/mL in LILRB1R-, −B1S-, −B3R-, and −B3S-transfected cells lines, compared to the mock control, respectively (Figure 7). The expression level of protein and mRNA transcripts for Th17 cytokine (IL-17A and IL-12p40) showed similar patterns with those of IFN-γ (Figure 7). The results indicate that LILRB1R- and LILRB3R-transfected cells produced more IFN-γ, IL-17A, and IL-12p40 proteins than LILRB1S- and LILRB3S-transfected cells.

## DISCUSSION

In this study, we identified and characterized novel *LILRB1* and *B3* genes from two genetically disparate chicken lines using a bioinformatics approach and *in vitro* analysis. Our initial analysis yielded 4 group transcripts as potential *LILRB1* and *LILRB3* genes from chicken lines 6.3 and 7.2, which were then analyzed using phylogeny alongside other structurally similar Ig-superfamily receptors. Comparison of amino acids identities and similarities between LILRB1 and LILRB3 in line 6.3 to those proteins in line 7.2 ranked between 97%–99%, and also between LILRB1 and LILRB3 to the corresponding mammalian proteins ranked between 13%–19% and 13%–69%, respectively. Moreover, phylogenetic analysis displaying amino acid similarity placed the LILRB1R, −B1S, −B3R, and −B3S together with members of the *LILRB1* and *LILRB3* genes of mammals suggesting that the 4 receptors are closely associated with *LILRB1* and *LILRB3* genes from other species. Together with the high degree of sequence similarity, this indicates that the LILRB1R, −B3R, and −B1S, −B3S amino acids isolated from the disease-resistant line 6.3 and susceptible line 7.2, respectively, represent homologs of the novel receptor in the chicken LILRB family. Moreover, the human LILRB1 and LILRB3 receptors contain 4 canonical and permissive ITIMs with NxYxxV, VxYxxV, VxYxxL and SxYxxL being present on every receptor [5], whereas the chicken LILRB1R, −B1S, −B3R, and −B3S receptors only contain one canonical ITIMs (LxYxxL) on every receptor. This possibly provides a greater diversity of signaling abilities by human LILRB1 and LILRB3 than that of chicken *LILRB1* and *LILRB3* genes. Our findings indicate that the *LILRB1* and *LILRB3* genes can be subdivided into two groups: transmembrane molecules with two ITIM motifs (LxYxxL and SxVxxV) in the resistant line 6.3 and two ITIM motifs (LxYxxL and LxYxxV) in the susceptible line 7.2. On the other hand, LILRB1R and −B1S have two Ig domains whereas LILRB3R and −B3S contain only one Ig domain (Figure 1C). The results may be implicated in the diversity of signaling abilities for *LILRB1* and *LILRB3* genes in resistant line 6.3 and susceptible line 7.2. In addition, at least one of these motifs contains the sequence V/YxxL/V, which has been shown to be the consensus sequence for binding by the SH2 domains of the SHP-1 or SHP-2 phosphatase. Another report had shown that tyrosine-phosphorylated LILRB1 associates and coimmunoprecipitates SHP-1 and SHP-2 [23]. Evidence from other immunoreceptors suggests that the phosphorylation of such motifs generates binding sites for other signaling molecules, including the SHIP [23]. To demonstrate this point, we analyzed the *LILRB1* and *LILRB3* genes-transfected cell from two chicken lines associated with SHP-2 by immunoprecipitation, FACS analysis, and qRT-PCR. The results indicate that the *LILRB1* and *LILRB3* genes-transfected cell in the two chicken lines associated with SHP-2 was significantly higher in LILRB3-containing cell line than in LILRB1-containing cell line. In addition, they were higher in the chicken line 6.3 than in line 7.2. Recently, some researchers suggested that SHP-2, a cytoplasmic SH2 domain-containing protein tyrosine phosphatase, is involved in the signaling pathways of a variety of growth factors and cytokines such as JAK-STAT and MAPK signaling pathway, and plays an important role in transducing the signal from the cell surface to the nucleus, and is a critical intracellular regulator of cell proliferation and differentiation [4]. Taken together, *LILRB1* and *LILRB3* genes in the two chicken lines could differentially regulate the signaling pathways and cytokines production.

In humans, the binding of LILRB1, but not LILRB3, to HLA-A, -B, and -C, and other non-classical class I molecules such as HLA-E, HLA-G, and HLA-F, is currently being evaluated in several cell types [2]. There are at least two predicted LILRB1 and LILRB3 ligands for each of the *LILRB1* and *LILRB3* genes, and most of the ligand candidates of these genes bind to MHC class I and β2m antigens. Moreover, these genes are believed to have other ligands or binding residues that differ in the two genetically disparate chicken lines.

By immunoprecipitation, FACS analysis, and qRT-PCR, it was confirmed that *LILRB1* and *LILRB3* genes in the two chicken lines strongly bind to MHC class I and β2m. The LILRB1 and LILRB3 proteins binding to MHC class I was significantly higher than those binding to β2m. In addition, the *LILRB1* and *LILRB3* genes-transfected cell binding to MHC class I and β2m in line 6.3 were markedly higher than those in line 7.2. Moreover, *LILRB1* and *LILRB3* genes significantly upregulated MHC class I-related genes such as TAP-1 and TAP-2, which are essential for antigen presentation by the MHC class I pathway, by more than 100-fold. The finding that MHC class I is recognized by inhibitory receptor genes LILRB1 and LILRB3 in two genetically disparate chicken lines expressed on macrophage cells expands the concept of self-recognition in immune response. Taken together, our results show that *LILRB1* and *LILRB3* genes are necessary and sufficient for the induction of MHC class I expression. Future analyses of the *in vivo* function of *LILRB1* and *LILRB3* genes are required to reveal if these two molecules play redundant or more exclusive roles in MHC class I-dependent immune responses.

A deeper understanding of the intracellular signal transduction pathways initiated by *LILRB1* and *LILRB3* genes is necessary to know how these genes can activate the expression of immune-related genes. Concurrent with a previous report showing that JAK/STAT pathway plays a key role in cytokine-induced biological responses, we demonstrated the effects of *LILRB1* and *LILRB3* genes on the JAK/STAT signaling pathway in the activated HD11 cell line [24]. Moreover, LILRB groups have been shown to regulate TLR activity [8], antigen-presenting phenotype and cytokine production [8]. Thus, the LILRB groups play an important role in the regulation of innate immune responses. The expression levels of phosphorylated STAT1 (p-Ser^727^), STAT3 (p-Ser^727^), and JAK2 (p-Tyr^1007^/Tyr^1008^) molecules and un-phosphorylated (STAT1/3, JAK2, TYK2, and SOCS1) molecules in response to *LILRB1* and *LILRB3* gene-transfected HD11 cell line compared to mock control by western blotting were increased, and also significantly upregulated in STAT1/3, JAK2, TYK2, and SOCS1 mRNA by qRT-PCR. Our results indicate that LILRB1 and LILRB3 activate and regulate the JAK/STAT signaling pathway and also control the immune system.

This study provides the first evidence indicating the possible role of JAK/STAT pathway in the signaling mechanism of chicken *LILRB1* and *LILRB3* genes in the chicken HD11 cell line. To investigate the function of *LILRB1* and *LILRB3* genes for growth factor activation, 3 chemokine (*CCL-4*, *CXCL-13*, and *CXCL-14*) and 12 cytokine (*IL-1β*, *IL-4*, *IL-6*, *IL-10*, *IL-12p40*, I*L-17A*, *IL-17F*, *IFN-α*, *IFN-β*, *IFN-γ*, *LITAF*, and *TGFβ4*) genes in the immune cell were measured by qRT-PCR. The expression of these cytokine genes was increased in LILRB1 and LILRB3-transfected cells of line 6.3 than those of line 7.2. As previously reported, chemokines appear to promote host resistance by mobilizing leukocytes and activating immune functions that kill, expel, or sequester pathogens [25] and IFN-α, IFN-β, IFN-γ, and IL-17 families play important roles in conferring resistance against pathogens. They also increase the expression of class I MHC molecules on cells making them more resistant to pathogens and are highly produced from Th1 and Th17 cells in response to pathogens [26]. IFN and IL-17 induce *IL-1β*, *IL-6*, *LITAF* [27] and *TGFβ4* mRNA production [26]. Moreover, Th2 cytokine (IL-10) and Treg cytokine (TGFβ4) mRNA have been reported to upregulate the cell surface expression of the inhibitory receptors on monocytes and dendritic cells and these mRNA also associated with the LILRB group in response to pathogens such as HIV [28]. Our result indicates that chicken *LILRB1* and *LILRB3* genes activate the JAK/STAT signaling pathway, and upregulate the expression of chemokines such as Th1, Th2, and Th17 cytokines. Expression levels of chemokines and cytokines were significantly higher in LILRB1- and LILRB3-transfected cells of the resistant line 6.3 than those of the susceptible line 7.2. Therefore, we demonstrated that *LILRB1* and *LILRB3* genes can induce several chemokines, such as Th1, Th2, and Th17 cytokine mRNA, which may be essential in the pathogenesis of diseases, highlighting the importance of this novel pathway in chicken disease.

In summary, this is the first report of the cloning, structural analysis, and function of the novel chicken *LILRB1* and *LILRB3* genes isolated from two genetically disparate chicken lines. Like other LILRB family members, *LILRB1* and *LILRB3* genes possess ITIM motifs in the cytoplasmic domain that bind to cytosolic tyrosine kinases such as SHP-2. We also showed that the *LILRB1* and *LILRB3* genes bind to MHC class I and β2m as well as other genes involved in class I antigen presentation, processing and regulation of immune responses. Moreover, *LILRB1* and *LILRB3* genes induce and regulate the JAK-STAT signaling pathway and upregulate Th1, Th2, and Th17 cytokine genes in the chicken cell line.

## Supplementary Data



## Figures and Tables

**Table 1:** Similarity (upper) and identity (below) of *LILRB1* and *LILRB3* genes from two genetically disparate chicken lines and other species

Species	Genes	B1R	B1S	B1	B1	B1	B1	B3R	B3S	B3	B3	B3	B3	B3	GenBank Acc.

		Chicken		Human	Chimp	Mouse	Pig	Chicken		Human	Chimp	Mouse	Monkey	Pig	
Chicken	*B1R*	-	99.10	38.35	35.20	37.23	67.37	79.97	79.97	38.24	38.24	15.74	32.28	69.85	RNA-Seq
Chicken	*B1S*	97.53	-	38.24	35.09	37.12	67.37	80.65	80.65	38.13	38.13	15.63	32.17	70.30	RNA-Seq
Human	*B1*	18.15	17.90	-	89.98	85.37	43.64	35.88	35.88	80.98	81.32	43.41	75.36	39.93	NP_001265327
Chimpanzee	*B1*	18.46	18.20	89.90	-	86.72	41.16	33.40	33.29	78.51	79.86	42.74	76.37	39.25	NP_001008979
Mouse	*B1*	17.23	16.97	79.02	82.60	-	43.86	35.65	35.54	78.51	79.52	43.19	74.12	40.71	NP_001035762
Pig	*B1*	13.97	13.66	34.78	35.09	35.71	-	65.69	65.57	43.41	43.98	19.12	37.68	69.85	XP_013838319
Chicken	*B3R*	45.53	47.22	16.76	17.07	17.07	13.66	-	99.21	36.22	36.44	12.93	30.03	68.72	RNA-Seq
Chicken	*B3S*	45.23	46.91	16.76	16.76	16.76	13.35	97.86	-	36.33	36.44	12.82	30.03	68.84	RNA-Seq
Human	*B3*	16.61	16.35	70.72	70.56	68.98	33.54	15.85	15.85	-	93.70	48.70	84.36	41.73	NP_001074919
Chimpanzee	*B3*	16.61	16.35	71.94	72.90	69.88	34.16	16.46	16.15	90.49	-	49.26	84.36	41.61	NP_001009055
Mouse	*B3*	18.76	18.51	49.05	47.58	47.17	29.81	15.54	15.24	53.79	54.83	-	46.56	17.32	AAH26937
Monkey	*B3*	16.61	16.35	71.13	69.78	69.43	34.47	15.24	15.24	84.17	85.26	49.19	-	35.20	XP_005590358
Pig	*B3*	16	16.66	26.76	27.69	25.23	18.32	15.38	15.69	25.84	25.23	24	25.53	-	XP_013854226

*LILRB*, leukocyte immunoglobulin-like receptors.

The nucleotide sequence of *LILRB1* and *LILRB3* genes were identified from RNA-Seq data in spleen and intestinal mucosa layer of two genetically disparate chicken lines afflicted with necrotic enteritis.

**Table 2:** Candidate binding ligands of *LILRB1* and *LILRB3* genes in two genetically disparate chicken lines

Name	Ligand	Binding residues	Domain	p-value	Binding with	References
LILRB1R	ZN	A^51^ Q^52^ G^128^ P^131^ G^133^ S^135^	1–151	3.12E-07	MHC class I	[30]
	EDO	H^185^ K^186^ D^187^ T^218^ Y^219^ R^220^	152–355	6.25E-06	MHC class I, β2m	[31,32]
LILRB1S	ZN	Q^52^ G^128^ L^129^ Q^130^ P^131^ A^132^	1–151	5.10E-07	MHC class I	[30]
	EDO	H^185^ K^186^ D^187^ T^218^ Y^219^ R^220^	152–355	7.69E-06	MHC class I, β2m	[31,32]
LILRB3R	EDO	L^30^ P^32^ S^33^ Q^34^ E^111^ L^112^ V^113^	1–120	1.14E-07	MHC class I, β2m	[31,32]
	NAD	L^164^ G^200^ Q^202^ E^208^ L^209^ S^210^ R^211^ P^213^	121–247	2.03E-04		
	PRX	V^278^ P^279^ L^280^ C^281^ L^282^	248–328	3.18E-02		
LILRB3S	KDO	W^54^ W^56^ Y^68^ H^96^ V^97^ S^98^ L^101^	1–120	4.98E-08	MHC class I, β2m	[33]
	IDO	G^145^ M^146^ M^217^ G^218^ Q^321^ L^322^	121–328	8.54E-03		

*LILRB*, leukocyte immunoglobulin-like receptors; MHC, major histocompatibility complex; ZN, zinc ion; EDO, 1,2-ethanediol; NAD, nicotinamide-adenine-dinucleotide; PRX, adenosine-5′-monophosphate-propyl ester; KDO, 3-Deoxy-D-mano-oct-2-ulosonic; IDO, iodide.
