# Supplementary material for: Chicken novel leukocyte immunoglobulin-like receptor subfamilies B1 and B3 are transcriptional regulators of major histocompatibility complex class I genes and signaling pathways
Source: Asian-Australas J Anim Sci. 2018 Oct 26;32(5):614–28. doi: 10.5713/ajas.18.0561 (PMC6502725; doi:10.5713/ajas.18.0561)
Supplement: Supplementary file 1 [file ajas-18-0561-suppl.pdf]

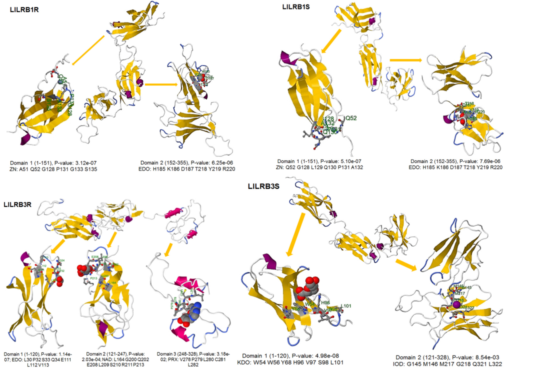

Supplementary Figure S1. Prediction of candidate ligands of LILRB1 and LILRB3 genes from two genetically disparate chicken lines. The structure and ligand-binding sites of the proteins were determined by molecular replacement using the program RaptorX web server. To identify the function, the ligands of LILRB1 and LILRB3 genes were mapped and searched in the RCSB protein data bank (http://www.rcsb.org).

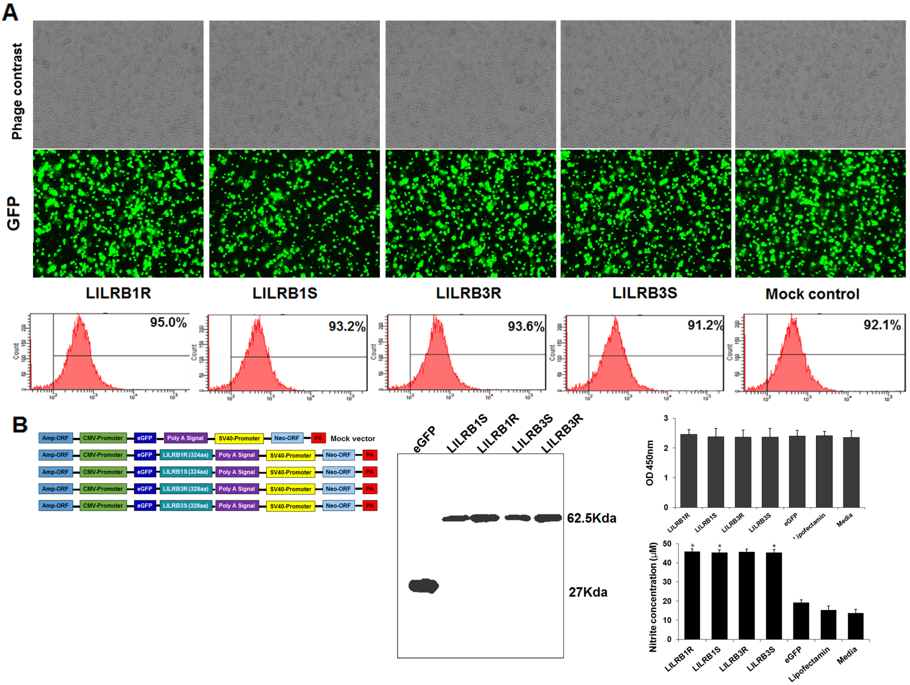

Supplementary Figure S2. Subcellular distribution of chicken LILRB1 and LILRB3 genes. (A) HD11 cells were transiently transfected for 48 h with an expression plasmid-encoding chicken LILRB1 and LILRB3 fused to GFP, and FACS analysis was conducted on GFP-positive cells (bottom). (B) Schematic representation of the LILRB1 and LILRB3 gene expression constructs (left); CMV = human cytomegalovirus immediate early promoter; ORF = open reading frame; PolyA = bovine growth hormone polyadenylation site. Western blot analysis was performed with antibodies against GFP [29] and cytotoxicity of LILRB1R, -B1S, and -B3S linked eGFP vector transfected into HD11 cell line after 48 h transfection was determined using cell proliferation and NO production assay.

Table S1. Depicts the gene ID, transcript ID, sequence length, location and expression of chicken LILRB by RNA-Seq.

Genes ID	Transcript ID	Name	Chr	Start	End	bp	NE-afflicted		Control	
							Line 6.3	Line 7.2	Line 6.3	Line 7.2
MERGEG028618	CUFF.25157	LILRB1	Un_random	46363100	46365527	2427	3.29	0.68	0.10	0.02
MERGEG029467	ENSGALG00000017594	LILRB1	Un_random	58593441	58594933	1492	9.14	2.04	1.02	0.22
MERGEG029546	ENSGALG00000022003	LILRB1	Un_random	59460063	59462402	2339	39.91	48.52	3.29	0.67
MERGEG025678	ENSGALG00000022592	LILRB1	Un_random	3559608	3565149	5541	10.26	2.48	0.39	0.07
MERGEG025875	ENSGALG00000022569	LILRB3	Un_random	6507193	6510564	3371	1.32	0.55	0.17	0.06
MERGEG026424	ENSGALG00000018513	LILRB3	Un_random	13670278	13671191	913	9.64	7.29	0.53	0.14
MERGEG026489	ENSGALG00000022506	LILRB3	Un_random	14574334	14576271	1937	2.39	0.38	0.25	0.01
MERGEG028804	CUFF.25323	LILRB3	Un_random	49437244	49439066	1822	14.42	3.00	0.61	0.16
MERGEG029015	ENSGALG00000012983	LILRB3	Un_random	52143047	52144313	1266	28.74	2.53	2.34	0.37
MERGEG029587	ENSGALG00000021997	LILRB3	Un_random	60091116	60092727	1611	37.35	7.59	3.75	0.57
MERGEG029837	ENSGALG00000018604	LILRB3	Un_random	63670814	63671960	1146	22.23	12.67	2.65	0.72

Table S2. Primer sequences for cloning and real-time PCR analyses of cytokine expression levels.

Primer	F/R	Nucleotide sequence (5′-3′)	Accession No	Note
GAPDH	F	TGCTGCCCAGAACATCATCC	NM_204305	qRT-PCR
	R	ACGGCAGGTCAGGTCAACAA		
IFN-γ	F	AGCTGACGACGGTGGACCTATTATT	HQ739082	
	R	GGCTTTGCGCTGGATTC		
IL-6	F	CAAGGTGACGGAGGAGGAC	JQ897539	
	R	TGGCGAGGAGGGATTTCT		
IL-17A	F	TGTCTCCGATCCCTTGTTCT	AM773756	
	R	GTCCTGGCCGTATCACCTT		
IL-17F	F	CTCCGATCCCTTATTCTCCTC	NM_204460	
	R	GTCCTGGCCGTATCACCTT		
LITAF	F	TGTGTATGTGCAGCAACCCGTAGT	AY765397	
	R	GGCATTGCAATTTGGACAGAAGT		
TGF-β4	F	CGTGCCCGTACATCTGGAG	JQ423909	
	R	GAGGGGGTCGAGGGTCTG		
IL-1β	F	TCGGGTTGGTTGGTGATG	NM_204524	
	R	TGGGCATCAAGGGCTACA		
IL-12p40	F	AGATGCTGGCAACTACACCTG	NM_213571	
	R	CATTTGCCCATTGGAGTCTAC		
IFNα	F	AACCACCCACGACATCCTTC	NM_205427	
	R	CAAGCATTGCTCGAGGTGC		
IFNß	F	CTTGCCCACAACAAGACGTG	NM_001024836	
	R	GTGTTTTGGAGTGTGTGGGC		
IL4	F	AGCACTGCCACAAGAACCTG	NM_001007079	
	R	CCTGCTGCCgTGGGACAT		
IL10	F	CTGTCACCGCTTCTTCACCT	AJ621254	
	R	ACTCCCCCATGGCTTTGTA		
CCL4	F	CCCCTTGTCATCGGTCAC	NM_204720	
	R	AGAGGCAGGAGCAGAGCA		
CXCL13	F	GCCTGTGCCTGGTGCTC	XM_420474	

	R	TGCCCCCTTCCCCTAAC		
CXCL14	F	GCCTTGCTTCTGCTGGTCATC	NM_204712	
	R	ATCTTATTTTCGGCCCTTTCCTT		
STAT1	F	TTGTAACTTCGCTATTGGTATTCC	NM_001012914	
	R	TTCCGTGATGTGTCTTCCTTC		
STAT3	F	AGGGCCAGGTGTGAACTACT	NM_001030931	
	R	CCAGCCAGACCCAGAAAG		
SOCS1	F	CTACTGGGGACCGCTGACC	NM_001137648	
	R	TTAACACTGATGGCAAAGAAACAA		
JAK2	F	CAGATTTCAGGCCGTCATTT	NM_001030538	
	R	ATCCAAGAGCTCCAGTTCGTAT		
TYK2	F	GCCCCATGCAGGAGGAAT	XM_427671	
	R	CTTTGCCACAGCCAGAATCAC		
SHP2	F	ATGTTGGTGGAGGGGAGAA	NM_204968	
	R	GGGGCTGCTTGAGTTGC		
β2m	F	CAGGTGTACTCCCGCTTCC	NM_001001750	
	R	GGCACGCCATCCTTCAT		
BF-I	F	GCACAGCCCCATCCTCT	AM279341	
	R	TGGCCCATCATTTTATTTCA		
BF-IV	F	CCATCCGGGGGTATTATCA	AF013492	
	R	TGGTGGGAACTGCCTCTG		
HLA-A	F	GGG ACT TCC TCG CCT TTG A	XM_003643799	
	R	ATC CCT TCC TCC TCC CAT CTC		
TAP1	F	GGCGGCTGCACACTACC	AJ843261	
	R	CCAGGGCTGAGAAACCACT		
TAP2	F	GACACCCGGCACCAGAT	NM_001099357	
	R	CCTCCTCGCCATTGAAGA		
LILRB1/3F	F	GCGGCCGCATGGCACCAATGGTGGTG	LILRB Transcript	Cloning
LILRB1R	R	CCTCTAGAGCGGGGTCCCCGCGTGC		
LILRB3R	R	CCTCTAGAGTGGGGTTCCCCAAAGCC		
